# Office-Based Intraosseous Infiltrations of PRGF as an Effective Treatment for Knee Osteoarthritis: A Retrospective Observational Clinical Study

**DOI:** 10.3390/jcm12134512

**Published:** 2023-07-06

**Authors:** Antonio Ríos Luna, Homid Fahandezh-Saddi Díaz, Manuel Villanueva Martínez, Roberto Iglesias, Roberto Prado, Sabino Padilla, Eduardo Anitua

**Affiliations:** 1 Department of Traumatology and Orthopedic Surgery, Clínica Orthoindal, 04004 Almería, Spain; iglroberto@gmail.com; 2 Department of Orthopedic Surgery, Hospital Universitario Fundación Alcorcón, 28922 Alcorcón, Spain; homidfsd@hotmail.com; 3 Department of Traumatology and Orthopedic Surgery, Avanfi Institute, 28015 Madrid, Spain; mvillanuevam@gmail.com; 4 Regenerative Medicine Laboratory, BTI—Biotechnology Institute I MAS D, 01007 Vitoria, Spain; roberto.prado@bti-implant.es (R.P.); sabino.padilla1958@gmail.com (S.P.); eduardo@fundacioneduardoanitua.org (E.A.)

**Keywords:** intraosseous, osteoarthritis, PRGF, platelet-rich plasma

## Abstract

The aim of this study was to explore and assess office-based ultrasound-guided intraosseous and intra-articular infiltrations of plasma rich in growth factors (PRGF) in patients with moderate and severe knee osteoarthritis (KOA). Seventy-nine patients (30 women and 49 men) with grade 3–4 KOA according to the Kellgren–Lawrence classification participated in the study. All patients were treated with a minimally invasive technique using local anesthesia WALANT (wide-awake local anesthesia no tourniquet) in the ambulatory setting. A PRGF intra-articular infiltration and two intraosseous infiltrations in the tibial plateau and femoral condyle were performed weekly for a total of three sessions. The evaluation of the results was carried out using knee injury and osteoarthritis outcome score (KOOS) at baseline and post-treatment. After a follow-up period of 11 months (median) [interquartile range, 7–14], all the KOOS domains showed statistically significant improvement (*p* < 0.001). Moreover, 88% of the patients showed a pain reduction of at least 10 points (minimally clinically important improvement) from pre- to post-treatment. Our retrospective study using the in-office procedure of ultrasound-guided combination of intra-articular and intraosseous infiltrations of PRGF is a safe and efficacious approach for the treatment of grade 3–4 knee osteoarthritis.

## 1. Introduction

Knee osteoarthritis (KOA) is a painful, chronic low-grade inflammatory disease in which the whole synovial joint tissue homeostasis is dysregulated [1], with the cartilage and synovium suffering the consequences of subchondral bone (SCB) biochemical disruptions in a loop manner among whole synovial joint tissues [1,2,3,4,5,6,7]. KOA is a highly prevalent disease, and the latest data suggest that it affects 37% of people over 60 years of age [8]. As a disease affecting the whole synovial joint tissues, the symptomatic treatment of KOA should target as many tissues as possible that are involved in the generation of pain, stiffness, and swelling that lead to mechanical failure, thereby limiting daily activities and quality of life [6,9].

Recently, mechanism-oriented therapeutic products and delivery strategies have emerged in the field of regenerative pain medicine to treat KOA pain and disabilities, among which autologous blood-derived products [10] are delivered via a dual approach by combining intra-articular and intraosseous routes [11,12]. Among these autologous biologics, plasma rich in growth factors (PRGF) is a type of platelet-rich plasma (PRP) with moderate enrichment in platelets and an absence of leukocytes and erythrocytes [13]. The immunomodulatory, anti-inflammatory, and antialgic effect of this bioresorbable and biocompatible versatile therapeutic tool is based on the gradual and progressive release of IGF-1, TGF-β, HGF, PDGF, and VEGF from the myriad of growth factors (GFs) contained in platelets and plasma. These biomolecules are trapped in the functionalized fibrin matrix after PRGF activation [14]. PRP, as well as isolated growth factors such as HGF, IGF-1, PDGF, and TGF-β which are contained in PRGF, have been reported to inhibit the NFkB inflammatory pathway in chondrocytes, synoviocytes, and macrophages [15,16,17,18,19], a common therapeutic target to dampen the expression of catabolic, proinflammatory, and senescent genes involved in osteoarthritis [20,21].

In line with this, a growing number of clinical studies have demonstrated favourable pain relief and improvement in the quality of life using a combination of intra-articular and intraosseous infiltrations of platelet-rich plasma (PRP) for the treatment of moderate and severe KOA [12,22,23,24,25]. This approach has been shown to be safe, with long-term clinical and functional benefits, and the procedure is easy to implement in the ambulatory setting using ultrasound guidance assisted with wide-awake local anesthesia no tourniquet (WALANT) technique, without sedation [26].

The aim of this study was to explore and assess this office-based ultrasound-guided intraosseous and intra-articular infiltrations of plasma rich in growth factors (PRGF) treatment in patients with moderate and severe KOA using the baseline and post-treatment knee injury and osteoarthritis outcome score (KOOS) as a means of evaluating the therapy.

Thus, the hypothesis of our study was that intraosseous and intra-articular infiltrations of PRGF improve pain and quality of life in patients with KOA on a clinically relevant basis.

## 2. Materials and Methods

### 2.1. Study Design and Data Source

This retrospective study was reported following the Strengthening the Reporting of Observational Studies in Epidemiology (STROBE) statement guidelines (Supplementary Table S1) [27]. The study protocol (AR-01-ER-22) was approved by the Institutional Review Board (CEIm-E) in accordance with the revised World Medical Association Declaration of Helsinki, amended in 2013 in Brazil [28]. In order to obtain the data for this study, a pseudonymized database of patients suffering from KOA treated with PRGF infiltrations in the Clínica Orthoindal between January 2020 and September 2022 was retrospectively reviewed.

The inclusion criteria for the study were as follows: (1) patients over 18 years old from both sexes; (2) patients diagnosed with grades 3–4 KOA according to the Kellgren–Lawrence classification; (3) patients treated with office-based intraosseous and intra-articular infiltrations of PRGF; (4) patients who completed the KOOS (knee injury and osteoarthritis outcome score) evaluation scale prior to PRGF intervention and at the end of the follow-up; and (5) a minimum follow-up of six months. The absence of complete data on the variables to be studied was the exclusion criterion.

### 2.2. PRGF Preparation

PRP was prepared according to PRGF-Endoret technology (BTI Biotechnology Institute, Vitoria, Spain). Briefly, peripheral venous blood was withdrawn into eight 9-mL collection tubes containing 0.4 mL of 3.8% (wt/vol) trisodium sodium citrate as an anticoagulant. Next, the blood was centrifuged at 580 g for 8 min at room temperature. The upper volume of the plasma was discarded. The 2 mL plasma fraction (F2), located just above the sediment red blood cells but not including the buffy coat, was collected. PRGF (F2) has a moderate enrichment of platelets and is free of leukocytes and erythrocytes, so it can be classified as pure PRP (P-PRP). Based on the aforementioned features of PRGF, and in accordance with the latest coding system [29], PRGF is a 24-00-11 PRP. In addition, Anitua et al. [13] recently published a comprehensive summary of the different classifications of PRGF. To initiate clotting and platelet activation following the instruction of the manufacturer, calcium chloride (10% wt/vol) was added to liquid PRGF F2 just before each infiltration (20 µL/mL PRGF).

### 2.3. PRGF Infiltration Procedure

The office-based intraosseous PRGF infiltration procedure was recently described by Rios et al. [26]. The entire treatment, including anesthesia and infiltration, was carried out on an outpatient basis (office-based procedures). Briefly, after blood extraction, the patient was positioned on the operating table in the supine position. Anesthesia was performed according to the WALANT technique and following the “hole-in-one” approach. A total of 10 mL of a mixture of 1% lidocaine (10 mg/mL) and epinephrine 1:100,000 (0.005 mg/mL) was prepared, followed by the addition of 1 mL of 8.4% sodium bicarbonate. This solution was infiltrated subcutaneously into the two entry points of the intraosseous infiltrations. The whole procedure was assisted with ultrasound guidance. The first infiltration was intra-articular, consisting of 8 mL of freshly activated PRGF and using the parapatellar external approach with a 21 G needle. Before proceeding with the intraosseous infiltrations, it was crucial to delay the injection of the local anesthesia by at least 30 min. Once the effectiveness of the anesthesia was assessed, drilling was performed with a 15 G trocar-biopsy needle system on the tibial entry. The trocar was placed 2 cm distal to the articular line with an inclination of 45° and 1.5 cm deep into the bone. Subsequently, 2 mL of the recently activated PRGF was gradually infiltrated via the trocar. The next step was the intraosseous infiltration of PRGF into the femoral condyle, with the trocar placed 2 cm proximal to the articular line with an inclination of 30° and advanced 1.5 cm deep into the subchondral femoral bone. Thus, 2 mL of the recently activated PRGF was infiltrated progressively via the trocar. The patient was able to easily get up from the operating table and begin walking immediately.

The complete treatment, namely, the intra-articular infiltration and the two intraosseous infiltrations in the tibial plateau and femoral condyle, was performed weekly for a total of three sessions.

### 2.4. Patient Evaluation and Follow-Up

The evaluation of the results was carried out using patient-reported outcomes. We used the validated KOOS (knee injury and osteoarthritis outcome score) scale, which was developed in the nineties and first published in 1998 by Ross et al. [30]. It is composed of five subscales that are measured separately: (1) KOOS pain (nine items); (2) KOOS symptoms with seven items, including other symptoms such as swelling, restriction of motion, and mechanical symptoms; (3) KOOS activities of daily living (17 items that measure the degree of involvement in activities of daily living); (4) KOOS sports/recreation (five items that reflect the degree of engagement at a more physically demanding level than daily life); and (5) KOOS quality of life (four items that measure the quality of life, social and mental aspects, such as lifestyle changes). A 5-point Likert scale was used for each item. Scores were converted to a 0–100 numeric scale, where 0 corresponded to extremely bad knee problems and 100 corresponded to perfectly healthy knees. Following the directions of Ross et al., an aggregate score was not calculated [30]. A reduction of at least 10 points on the KOOS pain scale, with respect to the pretreatment pain level, was considered a minimally clinically important improvement (MCII) [24]. Patients completed the KOOS questionnaire prior to treatment and at the end of follow-up, which, according to the inclusion criteria, was at least six months.

The safety of the procedure was also evaluated. All complications and adverse events were also gathered from the medical records of the patients.

### 2.5. Statistical Analysis

Descriptive data were presented as frequencies and percentages. The results of the questionnaires were presented as medians (interquartile range (IQR)). All data were tested for normality using the Shapiro–Wilk test. The box-and-whisker plots were generated following the Tukey style: the boxes show the median and the IQR, while the whiskers indicate the 25th percentile-1.5 × the IQR and the 75th percentile-1.5 × IQR. The round points outside the whisker range indicate the outliers [31]. The Wilcoxon matched-pairs signed rank test was used to observe changes in the KOOS domains pre- and post-treatment. Differences were considered statistically significant at a value of *p* < 0.05. Statistical analyses were performed using GraphPad Prism, version 9 (GraphPad Software, San Diego, CA, USA).

## 3. Results

### 3.1. Patient Characteristics

This study included 79 patients (30 women and 49 men), and 85 knees were treated with three weekly sessions of a combination of three intraosseous and intra-articular infiltrations of PRGF in the ambulatory setting assisted with the WALANT technique. The severity of KOA assessed by the Kellgren–Lawrence OA grade was grade 3 in 38 patients (44.7%) and grade 4 in 47 patients (55.3%). The median follow-up period was 11 months, with an interquartile range of 7–14 months. The baseline demographic characteristics of patients who participated in this study are shown in Table 1.

### 3.2. Clinical Outcomes

**Figure 1:**
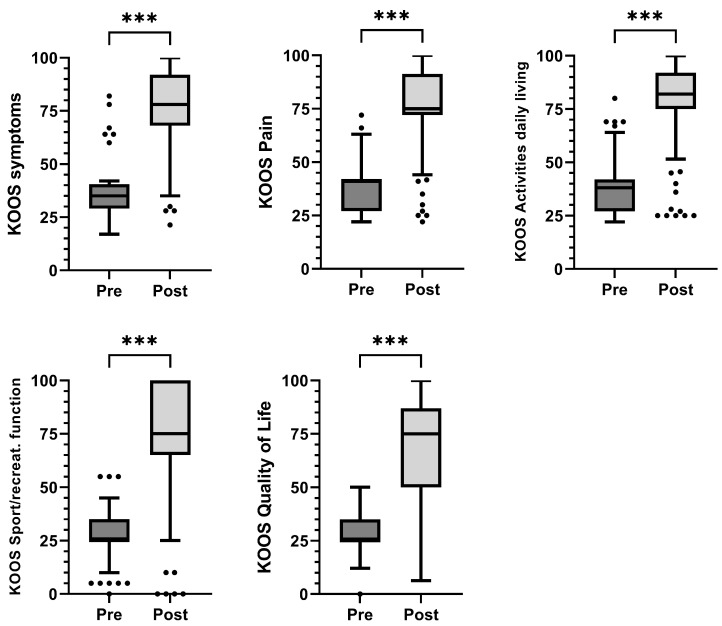
Results of the KOOS questionnaires. The results of the five subscales are shown graphically: KOOS symptoms, KOOS pain, KOOS activities of daily living, KOOS sports/recreation, and KOOS quality of life (*n* = 85 knees). *** indicates *p* < 0.001.

**Figure 2:**
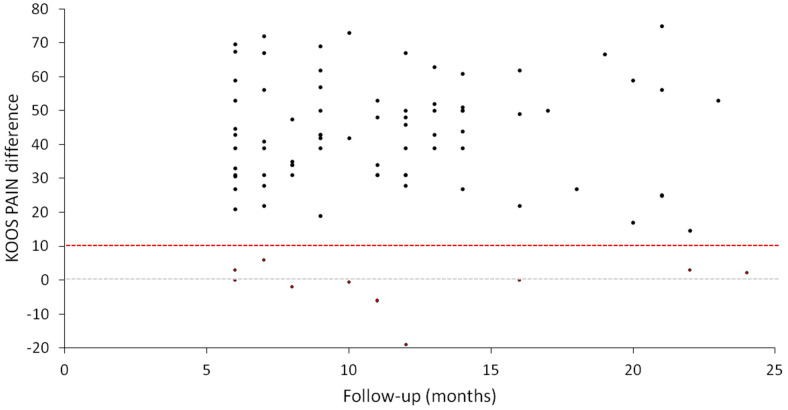
Graphical representation of the improvement in the KOOS pain scale versus follow-up time for the 85 knees included in the study. Seventy-five knees improved with treatment and exceeded the MCII for pain (black dots above the dotted red line), whereas only 10 knees (11.8%) were non-responders to treatment and did not reach this level of improvement (red dots). Each dot represents one knee in the study.

The assessment of patients after the follow-up period showed a statistically significant improvement in each of the domains of the KOOS questionnaire. Patients showed significant pain reduction (*p* < 0.001), symptoms (*p* < 0.001), activities of daily living (*p* < 0.001), sports/recreational activities (*p* < 0.001), and quality of life (*p* < 0.001) according to the KOOS score (Figure 1) (Table 2). The percentage of patients who showed pain reduction of at least 10 points (MCII) from pre- to post-treatment (6–24 months) was 88%, whereas 11.8% (10 knees) did not reach MCII improvement (Figure 2). It can be noted that the non-responders to treatment (10 knees) were evenly distributed throughout the follow-up period. Of the 10 knees that did not reach MCII, only 3 worsened, with a negative KOOS PAIN difference (Figure 2).

### 3.3. Adverse Events

Seven patients reported stiffness and pain during the first 24 h, two patients had knee swelling, and eight patients showed signs of inflammation at the entry points of the trocar. All these events were mended satisfactorily with oral pharmacological treatment with acetaminophen, which was allowed in the study.

## 4. Discussion

This study shows for the first time the clinical feasibility, safety, and efficacy of office-based ultrasound-guided intraosseous and intra-articular infiltration of the PRGF procedure in patients with grade 3–4 KOA. Patients improved significantly in each of the domains covered by the KOOS questionnaire after a median of 11 months of follow-up after PRGF treatment. The results of this study are in agreement with several clinical studies using intra-articular [32,33,34,35,36] or a combination of intraosseous and intra-articular PRP infiltrations and reporting a clinical improvement in pain and functionality of patients assessed by KOOS subscales [12,22,24,25,37], even delaying the need for knee arthroplasty between 1.5 and 5 years [23]. Compared to other treatments, two recent systematic reviews of level 1 evidence studies concluded that PRP is more effective than hyaluronic acid and at least as effective as bone marrow aspirate concentrate in the treatment of KOA [38,39].

By using the intra-articular route with this growth factor delivery system, PRGF reaches the synovial membrane (SM) and articular cartilage, two tissues that are sometimes inefficiently targeted by systemic drug delivery. Moreover, the intra-articular route circumvents systemic toxicity and its side effects, presents an excellent bioavailability, and does not entail molecular size limitation, in contrast to the systemically delivered molecules entering the joint via the capillaries of the subsynovium [40,41]. PRGF operates as a dynamic liquid scaffold with a fibrin network from which GFs are gradually released into the tissue, thereby overcoming the challenge of short joint dwell time of drugs, as the lymphatic drainage clears proteins within a few hours [42,43]. However, the role of the SCB in the pathophysiology of OA [44,45,46] is now well recognized, which might make the intra-articular route insufficient to tackle all the joint tissues involved in KOA. This realization has led to a new strategy to safely deliver PRGF to the damaged synovial joint by combining intra-articular and intraosseous infiltrations as an in situ biological ‘joint centric’ approach to treat osteochondral defects, osteochondritis dissecans, osteonecrosis, bone marrow edema-like lesions, and OA [11,44]. This “joint-centric” approach to treating severe knee OA conveys a pool of growth factors, including TGF-β, HGF, SDF-1, and IGF-1, naturally embedded in the cell-friendly scaffold of fibrin into four synovial joint tissues, namely, articular cartilage, synovial fluid, SM, and subchondral bone (SCB) [11,12,44]. The importance of treating KOA as a whole-organ disease has recently been demonstrated by the finding that the microchannel network of the subchondral bone is already modified and shows collective structural deterioration in the early stages of KOA [47]. Significantly, the cells and extracellular matrix of knee joint tissues are dysregulated, rendering SM and SCB a source of pain and inflammatory cytokines [7,10,48,49]. In this respect, the administration of PRP to the osteoarthritic knee and hip has been shown to exert antalgic and immunomodulatory effects [22,25,34,50,51,52], and it has been reported to modify the senescence phenotypes of mesenchymal stem cells (MSC) and osteogenic lineage at the subchondral bone level in mice and humans [53,54,55]. Importantly, the senescence of stromal cells in synovial joints has been associated with inflammation and pain [6,49]. In line with this, PRP might exert a senolytic activity on subchondral MSCs by restoring the osteogenic microenvironment and TGF-β homeostasis [56,57], thereby contributing to the attenuation or elimination of pain and even operating as a disease-modifying approach [49,58,59], but this remains to be confirmed by more histological and image-based evidence in humans. In addition, SDF-1, TGF-β, and fibronectin, which are part of the PRP biomolecules embedded in the fibrin matrix, exert chemotactic and chondrogenic effects and influence endogenous MSC homing from synovial fluid [50]. Accordingly, PRGF-like platelet-rich plasma containing a low platelet concentration and very few leukocytes has been shown to positively influence migration, proliferation, and chondrogenic differentiation of cultured human subchondral mesenchymal progenitor cells [60,61,62]. In line with this, IGF-I and -II, PDGF, SDF-1, TGF-β, CCL5, and fibronectin, all soluble morphogens trapped in the PRGF fibrin matrix, have been reported to influence recruitment and homing, and the chondrogenic differentiation of chondroprogenitor or MSCs from subchondral mesenchymal progenitor cells [61,63]. Finally, Sanchez et al. [44] suggested that the balanced ratio between platelet-secreted TGF-β1 and VEGF, and plasma growth factors such as IGF-1 and HGF, all conveyed by PRP intraosseous infiltrations, might reduce or buffer the excess of TGF-β in SCB and restore HGF activity synthesized by SCB cells [44]. This modulatory effect of PRP on the TGF-β1 signalling pathway might contribute to the shrinking of the fibroneurovascular tissue that replaces the bone marrow of OA subchondral bone. This fact might be explained by the antifibrotic mechanism already reported to be exerted by PRP on several cell phenotypes [64,65,66]. This new PRP-induced homeostatic balance between TGF-β1 and HGF [67,68] reduces the synthesis of NGF, VEGF, and other inflammatory mediators, thereby resulting in the modulation of pathologic fibroneurovascular tissue and the reduction of pain and hyperalgesia [69].

Importantly, this in-office procedure of intraosseous infiltrations with PRGF represents a step forward in the cost-effectiveness of the treatment of knee osteoarthritis that healthcare systems and surgeons should take into account as a significant therapeutic tool to safely treat moderate and severe KOA [70,71,72].

However, this study presents several limitations. The first limitation is inherent in the study design, as this was a retrospective study based on the usual clinical practice. Another limitation was the mid-term follow-up of 11 months. In order to consider this treatment as a disease-modifying approach, the follow-up should have been longer and time-homogeneous for all the patients participating in the study. However, it was reported that bone marrow lesions disappeared after this treatment [24,36], and several in vivo studies reporting structural modifications after PRP administration reinforce this potential effect [55,59,73]. Another drawback is the absence of a control group, as it has been shown that intra-articular treatment entails a placebo effect [74]. A third limitation is the lack of X-ray or MRI studies, which would have been very useful to confirm eventual changes in the SCB after ultrasound-guided intraosseous and intra-articular infiltrations of PRGF.

## 5. Conclusions

In summary, considering the limitations of this retrospective study, this study shows that intra-articular and intraosseous PRGF infiltrations performed in an office setting are safe and effective in the treatment of moderate-to-severe KOA. Further randomized clinical trials are needed to confirm the data obtained in the present usual clinical practice study.

## Figures and Tables

**Table 1:** Baseline demographic characteristics of patients.

Knees (*n*)	85
Patients (*n*)	79
Gender	
Female (*n*, %)	30 (38.0%)
Male (*n*, %)	49 (62.0%)
Age (years, mean ± SD)	60.9 ± 9.6
Height (cm, mean ± SD)	170.3 ± 9.0
Weight (kg, mean ± SD)	80.0 ± 17.6
Body mass index (kg/m^2^, mean ± SD)	27.5 ± 5.2
Kellgren and Lawrence OA grade (*n*, %)	
Grade 3	38 (44.7%)
Grade 4	47 (55.3%)
Treatment side (*n*, %)	
Right (*n*, %)	55 (64.7%)
Left (*n*, %)	30 (35.3%)
Follow-up period (months, median (IQR *))	11 (7–14)

* IQR, interquartile range.

**Table 2:** Comparison of changes in KOOS.

	Pre-Treatment	Post-Treatment	*p* Value *
Domain			
Pain	41 (27–42)	75 (72–91.4)	<0.001
Symptoms	35 (29–40.5)	78 (68–92)	<0.001
Activities of Daily Living	38 (27–42)	82 (75–92)	<0.001
Sports/recreation	25 (25–35)	75 (65–100)	<0.001
Quality of life	25 (25–35)	75 (50–87)	<0.001

Values are expressed as median (interquartile ranges). * Wilcoxon matched-pairs signed rank test.

## Data Availability

All the obtained data used to support the findings of this study are available from the corresponding authors upon reasonable request.

## Supplementary Materials

The following supporting information can be downloaded at: https://www.mdpi.com/article/10.3390/jcm12134512/s1, Supplementary Table S1: Strengthening the Reporting of Observational Studies in Epidemiology (STROBE) statement checklist.

## Abbreviations

IQR	Interquartile range
KOA	Knee osteoarthritis
GFs	Growth factors
KOOS	Knee injury and osteoarthritis outcome score
MCII	Minimally clinically important improvement
MSC	Mesenchymal stem cells
PRGF	Plasma rich in growth factors
PRP	Platelet-rich plasma
SCB	Subchondral bone
SM	Synovial membrane
WALANT	Wide-awake local anesthesia no tourniquet
